# Dental Education

**Published:** 1902-03-15

**Authors:** Joseph Griffiths


### DENTAL EDUCATION.

BY DR. JOSEPH GRIFFITHS.

Read at the meeting of the International Dental Federation, at Cambridge, Eng.

*Sir Michael Foster and Gentlemen:*—I feel this is an occasion for introducing what I am about to say with apologies, because neither am I a dentist nor indeed do I know, except in very general terms, anything of the education of a dentist. But, as I have been asked to speak upon the subject now under consideration, I beg you will grant me that indulgence which you, tried by meetings and speeches during the last week, must be pretty well accustomed to exercise, and which I trust you will freely bestow upon me and the matter of my remarks. As, however, I represent the sister art of Surgery in this university and am engaged in the teaching of the art, as well as the science upon which the art is built up, I may be allowed to have a small say in the matter of the education of the dentist. This subject is not new to me, thanks to Dr. Cunningham, who has on many occasions brought it before me for discussion on the main principles which should guide in the bringing up of the dentist. As I have said above, I know nothing of the work of the dentist except in a general sort of way, which I gather on the occasions when I seek his advice and aid, and all I can hope to bring to the discussion of this interesting subject is the *point of view* of the surgeon, who is in one sense the father of the dentist, he being even now capable by law of undertaking if he chooses the practice of dentistry.

Now, I believe I am correct in making the following statement, that the dentists are divided among themselves as to the best means to adopt whereby they themselves can be best educated; and, broadly speaking, they are divided into two sections. To both sections, however, the desire to produce the best dentist is common, and each section naturally thinks it has found the right way. So far as I am able to gather and understand, this difference between the two sections may be expressed in the following manner: One section desires that every dentist shall be trained as a medical man is, and then take up dentistry; whereas the other section desires that a dentist shall be trained to his own profession from first to last. (Before we proceed any further, I think it would be well for me to state that all I am about to say applies to the average, and not to the exceptional dentist.) To emphasize this proposition, let us put it thus: One section desires dentists to be qualified medical men who have, as it were, taken up dentistry as an afterthought, and the other wishes for a dentist from start to finish. According to the former, the man would be given a general medical education, and it can be estimated at nothing more, to base his future practice of dentistry upon; whereas according to the latter he would be given an education upon which his future work directly depends.

Is the educution of a dentist to be that of a medical man with dentistry added on, or is it to be designed to meet his own requirements? is the question of the hour.

To help in the solution of this interesting problem, a brief comparison between the training of the medical man and that of the dentist may not be out of place. In the case of a medical man the first half of his educational career is spent in gaining a complete knowledge of the normal man, and he takes biology, chemistry and physics as introductory subjects to anatomy and physiology. This is done in order to give him a better understanding of the structure of the body in detail and of the functions of its several organs and tissues. The second half is spent in acquiring all that is known of morbid changes and abnormal functions, and in a training in the physical examination of any and every part of the human frame. In the earlier half, then, he is trained in methods adopted in the different subjects for eliciting knowledge, and in the second he is directed to employ the methods with which he is already familiar to determine as far as possible the physical condition of any or every part of the body.

On the other hand, in the case of the dentist the first period is spent in acquiring knowledge of the nature and of the mechanical properties of certain materials and in the training to perform accurate work, which must be done, so I understand, to a nicety—a training similiar to that of a mechanician. In the second period he is directed to acquire a general knowledge of the structure of the body and of the functions of its several parts; a minute acquaintance with the teeth and the jaws, and of the diseases they are liable to; with the application of the methods, already familiar to him, of dealing with the teeth in their morbid states.

Such I believe to be a fair general statement regarding the training at the present time of a medical man and of a dentist. Let us contrast the requirements of these two. The medical man requires a knowledge of the minute structure and of the functions of the whole body, but the dentist only a knowledge of the minute structure of the teeth and of the jaws, and a general idea of the rest of the human frame. A medical man requires only a general, but sound, idea of mechanical work, but the dentist a thorough knowledge of it, so that he may be able to perform his work with accuracy. A medical man requires a detailed knowledge of all diseased processes and their known causes, but the dentist a particular knowledge of morbid processes as seen in the teeth and jaws, and only a general idea of the morbid processes observed in the remainder of the body. Such a review brings out pretty clearly that the educational career of the medical man does not coincide with that of the dentist except in a few particulars.

Even in anatomy and physiology, in which their work comes nearest together, the dental student requires that which will give him a sound understanding of the teeth and their connection with, and relation to, the remainder of the body, whereas the medical student should be familiar with the whole body. Of course, the more a dentist knows of the human body or of any other kindred subject the better he will be equipped generally, but not necessarily better furnished for the work of his own profession.

And if we go further and compare the surgeon with the dentist, we shall find that their work differs in a material degree. The dentist must possess many of the qualities that go to make a surgeon; he should have a quick perception, a keen insight, a sensitive touch, and be ever ready to act. But the skill of the dentist is largely, if not entirely, the result of that training in the mechanical department, so to speak, whereas the skill of the surgeon depends less upon mechanical training than upon accurate judgment to do enough and no more—for in hardly any operation is it necessary for him to make a physicist's measurements and to adhere to them. Mechanical training has indeed been neglected in the education of a surgeon, and hence it is that we often deplore the mechanical knowledge and the reasoning built upon its deficiency as displayed even by surgeons of repute. This has been neglected, I imagine, because that skill born of judgment has been estimated so highly. Now, with the dentist it is just the reverse, for who can conceive a dentist who is ignorant of mechanical work? but one without judgment may perhaps be occasionally met with. Thus the surgeon is at one end of the scale and the dentist is at the other, and doubtless it would be a good thing to improve them both, but in contrary directions.

Enough has, I hope, been said to point out that to make a dentist his training should be so arranged as to bring out his fitness for the work before him. A mechanical training of the best kind is essential to him, and must form the basis of his future work. In addition he requires a knowledge of the minute structure and of the function of the teeth, the material upon which he will have to bring his best mechanical skill to bear. I would therefore strongly urge you not to imitate the education of a medical student, but to continue on the lines which will train a dentist for his own profession from first to last, and to have a single purpose in view and to endeavor to obtain a definite result. Do not try to make a medical man a dentist, but let a dentist start and finish as such.

Can this education of a dentist be carried on side by side with that of the medical men? is the question of practical importance. I would unhesitatingly answer, No. The anatomist may train either, but he can not train both together without giving one much more than he requires and not paying enough attention to the other. It is much the same with physiology. Therefore, I say their courses should be separate, and so arranged as to serve the right end. In physics and chemistry the same training might serve; in study of diseases, No.

Is such a course of study proper for a university to undertake? In my humble opinion it is and should be, for the work of the dentist is as honorable and as worthy of respect as that of any of the older professions, and I trust that the newer universities will take this line and have an avenue for dental students to obtain a university degree side by side with the medical student. But I also trust the authorities will let dentistry and medicine be free to develop along those lines which each finds best suited for its own progress.

Although dentistry was once an intimate part of the medical art, it can hardly be so again, for its evolution has been so complete that it now forms a distinct and separate division of the art of healing. It is, I venture to think, a child of the old stock destined to continue an independent existence and to work out its own salvation.

DISCUSSION.

Dr. Brophy, Chicago, said that years ago he held views quite contrary to those he held to-day, but in the light of advancing education and the development of dentistry he had been forced to accept ideas that were formerly not agreeable to him. In the United States dentistry had its birth as a separate and distinct school of training; it was not from choice, but because the medical profession refused to give dentistry a place in its curriculum. At that time it was regretted, because it was wished that dentistry should be a part of the parent medical profession. But, independent schools having been founded, such branches of medicine were taught as the founders felt were necessary. The schools had now added, from time to time, departments, until one who was not acquainted with all the medical curriculum might readily be led to believe that they were schools of medicine. It was recognized that in Europe the conditions were quite different from those in America. It was felt that it was impossible to make changes in this country, and in many instances it was quite impossible to make changes in America. The question, therefore, was how to prepare a man to do his work. He had the pleasure of sitting by the side of Sir Michael Foster at luncheon, and I put to him this question, "I

have a boy nineteen years of age, just ready to enter the university; should he be prepared by a long four years' course in the university and then entered at the school of medicine for another four years' course, or not?" The answer was, "I would prepare him for his life's work. A student of medicine should devote his attention to the study of physics and chemistry and languages." Had he learned nothing more in his trip to Europe, he would have been fully compensated by that answer. He quite agreed with every word contained in the paper. The man who started out in life to prepare himself for any particular calling must have in mind that calling from the beginning to the end.

Dr. Sims Woodhead (professor of pathology, Cambridge), said that every man preparing for his life's work underwent a certain amount of general training, a training to fit him for the specific work he had to undertake later; and he could not help thinking that perhaps the preliminary years of the dental student and medical student might at any rate run on certain parallel lines. An attempt was made, how far successfully it was difficult to say, in the subject he had to deal with to give the medical student, as soon as he came from his study of anatomy and physiology, some inkling of the general processes of disease, The student specialized in the direction of special pathology, diseases of the nervous system, diseases of the kidney, and so on; but before he took this up it was absolutely necessary he should have a good solid foundation of the general processes of disease. That being the case, he could not help thinking it might be somewhat dangerous to begin to specialize at too early a stage, and whether it would not be better to study at any rate the general physiological and pathological processes, in order that in studying the disease of the special parts to which attention was to be devoted one might go to the very foundation straight away. From that point of view, he put in a plea for some common ground in the earlier part of the dentist's professional life. He recognized how much dental surgeons had contributed to the subject of bacteriology. In fact, the earliest experiments in bacteriology were carried out on material taken from the teeth. He quite agreed with dentists specializing in the latter stages of their course and not attempting to be medical men or surgeons, because they wished to treat a patient for special diseases of special organs, and it was their duty to know far more about those than any medical man or surgeon possibly could know. Dentists were experts, and therefore required an expert training. Even the surgeon found it necessary to specialize, and anything outside his own work he handed over to a colleague. In that light he hoped no attempt would be made to make the dental qualification a medical qualification, but that it would be made something far better for the purpose than any medical or surgical qualification. The dental surgeon was a man specializing in a certain direction, who had built up his profession on a good sound foundation of general physiological and pathological knowledge.

Professor Hesse, Leipzig, considered that the preparation for the dental profession was so different in different countries that it was almost impossible to fix a rule or a standard for the preparation in all countries. It was necessary to be guided by the point in view—namely, the development of the art or profession which it was intended to practice, and the training should be such as was best fitted to secure that end.

Dr. Kirk: It was said some years ago by a gentleman who resided not so many miles from this spot that "the evil that men do lives after them; the good is oft interred with their bones." But in what has taken place at this conference it seems to me we have at least one instance where the reverse of that proposition is true. If I have been able to correctly interpret the *motif* of the address by Sir Michael Foster I feel that I do him no injustice when I recognize in it the practical application of the principles set forth by Mr. Herbert Spencer in his epoch-making essay on Education; or when I further recognize in it the spirit which animated the life-work of that man, who, more than all others, I regard as the nestor of dental education in England, Sir John Tomes.

I was told before I left America, and even since my arrival in England, that it would be quite useless to expect that anything which might be done as a result of the conferences of the Federation would have auy effect in modifying existing views on the subject of dental education in Great Britain; and yet here in England there has been given out by one of her recognized educational authorities, and from one of her greatest universities, a statement of the principles of dental professional education the most liberal, logical and reasonable, which, in my judgment, has yet been uttered anywhere.

Like Mr. Spencer, Sir Michael Foster in his address recognizes the utilitarian character of professional knowledge and the inevitable conclusion therefrom, that education should, from the beginning, be adapted to the uses which the knowledge thereby attained is intended to subserve. He has, by keeping that central idea in view, cut the Gordian knot which for years has confused our discussions and thought on the relationship of dentistry to medicine, His statement that the dentist, within the limits of his activities, is a "healer" places the dental practitioner upon the basis of a natural classification much more readily understandable than when he is regarded, either positively or negatively, as a medical specialist, for, lacking as we do an adequate definition of medicine, it is not yet possible to decide whether a dentist is a medical specialist or not.

The enthusiasm and unanimity of appreciation with which the address of Sir Michael Foster has been received clearly indicate the general acceptance which this representative international gathering has accorded to the views he has expressed. So evidently is that the case that it seems to me the further deliberations of this body may be most profitably confined to a study of the dental curriculum, or, in other words, to securing an arrangement of professional study in conformity with the principles set forth in the address, best suited to the education of the dentist. The best attainable curriculum is yet to be devised; the very fact that such marked differences are to be found in the curricula of dental colleges throughout the world is self-evident proof of the need for further investigation of the best methods for making dentists. It would simplify the question greatly if we should arrange all of the subjects now taught in all dental colleges into two categories: First, those which are essential, and, second, those which, though not essential, are desirable in the education of the dentist. We would then be in position, after having formulated a minimum essential curriculum, to provide for its continued expansion and improvement by the gradual inclusion of members of the category of desirables into that of the essentials.

Reference has been made to the importance of manual training as a feature of the dental curriculum, in order that there may be given to the student that high degree of manipulative dexterity without which he is unable to achieve success as a dental operator. We all admit the importance of manual training in dental education, but not all dental educators have clearly recognized an equally important consideration in that connection—namely, the stage of development at which manual training should be undertaken by the student. I am not a physiologist, and I am glad to be in position to submit to the judgment of the distinguished physiologist, as well as educator, who is our presiding officer to-day, whether it is not true that in order to successfully train the hand to a high degree of dexterity the manual education must be undertaken early in life, for a period quickly arrives in later years where such training becomes impossible. It was that fact which was clearly recognized by Sir John Tomes, and which he so energetically and practically advocated in his efforts at dental educational reform in this country.

We have frequent and familiar examples of an analogous state of affairs in connection with the use of the bicycle. It is quite possible for an individual after attaining adult years to learn to ride the wheel, but the later it is put off the more certainly does the unfortunate rider develop that anxious expression of countenance which in America we call the "bicycle face." The learner may in time know how to propel his machine, but in so doing he acquires infinitely more knowledge about every feature of the topography of the roadbed over which he travels, and never acquires that freedom and abandon begotten of the automatic muscular coordination with which the street urchin of a dozen years controls his machine. It is the necessity for manual training for the dental student in an early period of his career, when his muscular and nervous receptivities are at their maximum, that we find, in my judgment, the strongest argument in favor of a special curriculum for the dental student, and a sufficient reason why his special education should not be deferred until after he has pursued a standard course of medical training.

Professor Griffiths has referred to the importance of including the study of bacteriology in the dental curriculum. I know of no better illustration of the practical utility of a knowledge of bacteriology to the operative dentist than that embodied in the statement recently made by Dr. Black, of Chicago, in which he said, with reference to the preparation of cavities in teeth preparatory to inserting fillings, that "the margins of all cavities should be laid down upon areas of tooth-structure which are relatively immune to the attacks of the bacteria which cause dental caries, in order to prevent a recurrence of the disease."

If that axiom be true, and I think that no one can successfully question its accuracy, the evident conclusion must be that no man can intelligently and successfully prepare a carious cavity in a tooth for filling except he be fortified by a fair knowledge of bacteriology. I feel that we may congratulate ourselves as dental teachers and practitioners upon our good fortune in securing the encouraging and far-sighted statement of dental educational principles embodied in the able address of our distinguished chairman and of those who have followed him.

Sir Michael Foster said, in answer to Dr. Kirk's inquiry, that for many years past he had urged that the education of the surgeon should not be delayed too long, because it was impossible after certain years to acquire that suppleness and dexterity of touch which was necessary for success. The mind grows old very slowly, and can be educated even late in life; but the body becomes old very soon, and it is necessary to train it while it is really young.

Sir James Crichton Browne was glad to have the opportunity of paying his tribute of admiration to the excellent address delivered that morning—an address instinct with wit and wisdom, adorned by epigrams and similes, which would not be easily forgotten. Sir Michael Foster referred to the early training of the dentist at the bench, and the subject had been further emphasized by the excellent observations of Dr. Kirk. Speaking from his own point of view, he attached great value and importance to the manual education of the dentist, and was inclined to attribute to that education a utility and significance that are not perhaps always generally recognized. Every surgeon knew that the movements of the hand were initiated in a certain group of centers of the middle region of the brain—motor centers of the brain. But they were motor centers only in a special sense. They were not motor simply in the sense of sending forth impulses in response to excitations from without; they were motor in the sense of being the springs of movement, and they were receptacles in which was chronicled all the knowledge which the muscular operations put the man in possession of. The muscles not only obeyed the commands of the will, but they added infinitely to the information and intellectual acquisitions. The most cursory analysis of ideas revealed the fact that there were very few of them which were known purely by sensory impressions. The motor centers of the brain took an enormous share in mental life, and mental manifestations would be as impossible without them as would be the circulation of the blood without one ventricle of the heart. The highest possible functional activity of the motor centers was as important with a view to mental power as to muscular expertness, and the motor centers for the hand were very prominent among the motor centers of the brain. They were related to an organ which in its enormous combination of movements largely added to our intellectual resources, and it was evident that the highest possible functions of activity of those centers was of value in adding to intellectual grasp as well as adding to the expertness of the hand and to business success. But in order to have the highest possible functional activity of those centers it was necessary to have them trained betimes, and therefore it was necessary to give the student his manual training in dexterity very early in life; and by doing that one was not merely training the hand, but was nelping to expand and develop the intellect.—*Cosmos*.

